# Genome-wide characterization of two *Aubrieta* taxa: *Aubrieta canescens* subsp. *canescens* and *Au. macrostyla* (Brassicaceae)

**DOI:** 10.1093/aobpla/plac035

**Published:** 2022-09-10

**Authors:** Yasin Kaya, Zübeyde Uğurlu Aydın, Xu Cai, Xiaowu Wang, Ali A Dönmez

**Affiliations:** Molecular Plant Systematic Laboratory (MOBIS), Department of Biology, Faculty of Science, Hacettepe University, Ankara 06800, Turkey; Molecular Plant Systematic Laboratory (MOBIS), Department of Biology, Faculty of Science, Hacettepe University, Ankara 06800, Turkey; Institute of Vegetables and Flowers, Chinese Academy of Agricultural Sciences, Beijing 100081, China; Institute of Vegetables and Flowers, Chinese Academy of Agricultural Sciences, Beijing 100081, China; Molecular Plant Systematic Laboratory (MOBIS), Department of Biology, Faculty of Science, Hacettepe University, Ankara 06800, Turkey

**Keywords:** Arabideae, *Arabis*, *Aubrieta*, Brassicaceae, genome evolution, whole-genome sequencing

## Abstract

*Aubrieta canescens* complex is divided into two subspecies, *Au. canescens* subsp*. canescens*, *Au. canescens* subsp. *cilicica* and a distinct species, *Au. macrostyla*, based on molecular phylogeny. We generated a draft assembly of *Au. canescens* subsp. *canescens* and *Au. macrostyla* using paired-end shotgun sequencing. This is the first attempt at genome characterization for the genus. In the presented study, ~165 and ~157 Mbp of the genomes of *Au. canescens* subsp. *canescens* and *Au. macrostyla* were assembled, respectively, and a total of 32 425 and 31 372 gene models were predicted in the genomes of the target taxa, respectively. We corroborated the phylogenomic affinity of taxa with some core Brassicaceae species (Clades A and B) including *Arabis alpina*. The orthology-based tree suggested that *Aubrieta* species differentiated from *A. alpina* 1.3–2.0 mya (million years ago). The genome-wide syntenic comparison of two *Aubrieta* taxa revealed that *Au. canescens* subsp. *canescens* (46 %) and *Au. macrostyla* (45 %) have an almost identical syntenic gene pair ratio. These novel genome assemblies are the first steps towards the chromosome-level assembly of *Au. canescens* and understanding the genome diversity within the genus.

## Teaser

*Aubrieta*is one of the ornamental rocky plants in the cabbage family (Brassicaceae) with an untouched genome. As initial research, the genome of morphologically intricate *Aubrieta canescens*subsp. *canescens*and *Au. macrostyla*was surveyed in terms of TEs, mutational diversity, orthologs and gene density. *Aubrieta canescens*subsp. *canescens*, and *Au. macrostyla*were assembled as ~165 and ~157 Mbp. In total, 32 425 and 31 372 gene models were predicted in the genomes, respectively. We used state-of-the-art computational tools to address the evolutionary genomics affinity of the taxa with some of the core Brassicaceae species with a comparison of *Arabis alpina*syntenic genes.

## Introduction

Brassicaceae is a moderately large, economically and scientifically important family comprising ca. 4000 species (Kiefer *et al.* 2014; Koch *et al.* 2018), including the model plant *Arabidopsis thaliana*, cabbage, turnip, radish, oil crops (e.g. *Brassica*, *Raphanus* and *Camelina*) and ornamentals (e.g. *Aubrieta*, *Arabis*, *Hesperis*, *Lobularia* and *Matthiola*) (Karl and Koch 2013; Nikolov *et al.* 2019). This family has one of the highest speciation rates among terrestrial plant groups. Because polyploidy has a major impact on plant evolution, some diversification in Brassicaceae can be linked to whole-genome duplication (Hohmann *et al.* 2015). Notably, the genus *Draba* has nine different ploidy levels, and the ratio of the number of taxa exhibiting polyploidy to the total number of taxa in the genus is 66 % (Jordon-Thaden and Koch 2008). Although three different ploidy levels were observed in the genus *Arabis* from the same tribe, Arabideae, the ratio of the number of taxa with polypoidy to the total number of taxa in the genus was 79 % (Koch *et al.* 2010).

*Aubrieta* belongs to the tribe Arabideae in the expanded lineage II, with a chromosome number of 2n = 16 (Al-Shehbaz *et al.* 2006; Beilstein *et al.* 2006; Franzke *et al.* 2011). Recently six clades (A, B, C, D, E, F) were identified for the family and the Brassicaceae lineage II was divided into three Clades B, C and D using the complete plastome (Couvreur *et al.* 2010; Huang *et al.* 2016; Guo *et al.* 2017); phylogenomic analyses produced a well-resolved phylogenetic tree including six major clades in Brassicaceae (Nikolov *et al.* 2019) but none of the *Aubrieta* taxa were included in these phylogenetic analyses.

The genus *Aubrieta* is difficult to classify taxonomically and is represented by 21 species that are distributed across the eastern Mediterranean region (Yüzbaşıoğlu *et al.* 2015; Dönmez *et al.* 2017; Koch *et al.* 2017). The endemic species, *Aubrieta canescens*, has traditionally been divided into three subspecies: *Au. canescens* subsp*. canescens*, *Au. canescens* subsp. *cilicica* and *Au. canescens* subsp. *macrostyla*. Of these, *Au. canescens* is a moderately diverse species of the genus *Aubrieta* and is distributed across three phytogeographical regions of Turkey. *Aubrieta canescens* subsp*. canescens* is distributed in Central Anatolia and its adjacent regions, *Au. canescens* subsp. *cilicica* is mainly distributed in the Taurus Mountains and *Au. canescens* subsp. *macrostyla* is distributed in eastern Taurus. Although the morphological characteristics show only low differentiation between taxa, the habitats of the species are quite different, which may indicate a physiological rather than a morphological level of differentiation. Recent molecular studies have indicated that *Au. canescens* subsp. *macrostyla* should be separated as a distinct species (Koch *et al.* 2017), and this taxonomic status is followed here. However, the *Au. canescens* complex requires further investigation to explore its phylogenetic relationship with the other taxa of the genus.

Members of the Brassicaceae family generally have small genomes, which allowed the sequencing of the first plant genome (Kaul *et al.* 2000) and the sequencing of the highest number of genomes to date (plant genomes database, http://www.plabipd.de/plant_genomes_pa.ep). In addition, the development of genomic techniques has greatly facilitated research into the largest crucifer tribe, Arabideae, and the genome evolution of the seven subclades of the tribe has been inferred from centromere repositioning (Mandáková *et al.* 2020). However, attempts to elucidate the genomic characters of the genus are limited and a publicly available genome assembly of *Aubrieta* has not yet been published.

Turkey is known to be one of the most important hotspots for native plants (Médail and Diadema 2009; Dönmez and Yerli 2018) and of genetic diversity centres of several crop plants (Zhukovsky 1951). All the complex taxa of *Au. canescens* are potentially ornamental plants and have been grown in numerous botanical gardens as rock plants.

In this study, we performed the whole-genome sequencing of *Au. canescens* subsp*. canescens* and *Au. macrostyla*, and their genome-wide characterization, including gene predictions, transposable element (TE) composition, variant identification and evolutionary comparisons.

## Materials and Methods

### Plant material, isolation and sequencing

*Aubrieta canescens* subsp. *canescens* (three accessions), and *Au. macrostyla* (three accessions) samples were used in this study. Seeds of the studied taxa were germinated in 2019, and one mature leaf originating from a single seed was selected for DNA extraction. Genomic DNA was extracted using a DNeasy *Plant Pro Kit* (Qiagen, Germany) and quantified using a Qubit fluorometer (Life Technologies, Foster City, CA, USA). Quality control was performed by analysing an aliquot of genomic DNA on an 1 % agarose gel. A paired-end genomic DNA library was constructed using the BGI DNB-seq platform and Macrogen company with a TruSeq DNA PCR-Free (350) kit. The library was run on NovaSeq, using a standard Illumina sequencing workflow **[see****Supporting Information—Table S1****]**.

### Genome assembly

Before assembly, adapters and low-quality reads were removed using SOAPnuke v2.1.6, (Chen *et al.* 2018) and quality control was performed using FastQCv0.11.9 (Andrew 2010). The optimum *k*-mer sizes were selected using the Kmerginie v1.7 software to match the reads correctly and rapidly (Chikhi and Medvedev 2014). Error correction of the raw data was performed using Karect v1.0 (Allam *et al.* 2015). Genome assemblies were constructed using *de novo* assembly and iterative mapping approaches. The genome assemblies were generated *de novo* using the de Bruijn graph approach on the SPAdes v3.15 (Bankevich *et al.* 2012) software with --careful and --only-assembler options. Subsequently, unmapped reads were iteratively mapped into the genome to correctly insert contigs that were mismatched or missing from other accessions using the BWA-mem v0.7.17 (Li 2013) algorithm. To create a less fragmented genome and close the gaps between contigs, we performed a post-assembly process using the Redundans v0.11 pipeline (Pryszcz and Gabaldón 2016). The assembly with the longest contig and the fewest number of contigs was selected according to the GAGE (GAGE: https://gage.cbcb.umd.edu/) criteria, for the rest of the downstream analyses.

### Reference-assisted chromosome scaffolding

Given the availability of a relatively good reference genome (*Arabis alpina* latest version retrieved from http://www.arabis-alpina.org) for *Au. canescens* (n=8) and *Au. macrostyla* (n=8), a reference-assisted scaffolding approach was used to optimize the genome. The scaffolds were aligned to the *A. alpina* genome using blastn74 in the blast+ toolkit 2.8.0-alpha (Madden 2013). These alignments were used by chromosomer v0.1.3 fragmentmap command to perform the chromosome scaffolding (https://github.com/gtamazian/Chromosomer) (Tamazian *et al.* 2016).

### Assessment of assemblies

An assessment of the draft genomes was performed using QUAST v5.0.2, with the default parameters (Gurevich *et al.* 2013). In addition, we investigated the completeness of genomes using the Benchmarking Universal Single-Copy Orthologs (BUSCO) v5.2.2 viridiplantae odb10 library (Simão *et al.* 2015). Additionally, Minimap2 v2.22 was used to map the raw reads onto the final assemblies to evaluate the accuracy of the assemblies (Li 2018).

### TEs and gene model prediction

To discover the *de novo* repeats in our assemblies, RepeatMasker (https://github.com/rmhubley/RepeatMasker) Repbase library and RepeatScout v1.0.5 software were used to predict TEs (Price *et al.* 2005). Gene prediction was performed on Augustus-ab-initio v3.3.0, using BLAST hints (70 % threshold) and protein sequences of *A. thaliana* and *A. alpina.* The coding DNA sequences were extracted using getAnnoFasta Perl script of Augustus (Stanke *et al.* 2004).

### Variant investigation

Polymorphism was determined by scanning all the loci across the genome of the *Au. canescens* complex. To accomplish this, the draft assemblies were indexed using the BWA-mem algorithm. The clean data of each genome were then mapped to assembled files. Reads were sorted by removing PCR duplications using the Samtools v1.12 markdup command (Li *et al.* 2009). The BAM files were indexed and prepared using the BamTools v2.5.2 index command to call the variants (Barnett *et al.* 2011). Haplotype-based variants were generated from BAM files using Freebayes v1.3.5 (Garrison and Marth 2012). The parameters used were as follows: minimum coverage value, 15; minor allele frequency, 0.05; and minimum base quality score, 20.

### Orthologs and synteny

A phylogenetic tree was constructed using the single-copy genes for *Au. canescens* subsp. *canescens* and *Au. macrostyla* and several other species representative of Clades A and B **[see****Supporting Information—Table S2****]**. Homologous genes between species were analysed using OrthoFinder v2.5.4 (Emms and Kelly 2019). A single-locus species tree obtained from orthologous sequences was revealed by a pairwise sequence similarity approach using STAG and STRIDE algorithms prepared by OrthoFinder (Emms and Kelly 2017, 2018). To reconstruct the phylogeny, multiple sequence alignment was first performed using the MAFFT algorithm, followed by the construction of species trees using FastME in the OrthoFinder software. Diversification times were based on gene similarity calculations derived from the ancestral genes. We used the Satsuma v2.0 software to compare the syntenic genes of genomes with its closest relative, *A. alpina* (Grabherr *et al.* 2010). We set the gene identity ratio to >70 % and the minimum coverage as 15×. After identifying the syntenic regions, we used Blastp and Blastn (NCBI-BLAST package, ftp://ftp.ncbi.nih.gov/blast/) to estimate the syntenic gene pairs in each genome. We used D-GENIES to visualize the assembled synteny (Cabanettes and Klopp 2018).

## Results and Discussion

### Genome assembly

**Figure 1.**
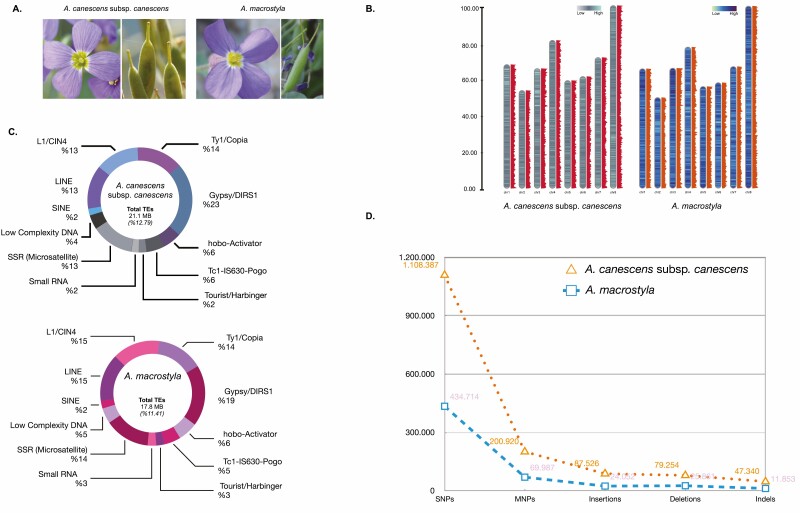
General habit and comparative gene density, repeat composition and polymorphism diversity across the genome of *Aubrieta canescens* subsp*. canescens*, and *Au. macrostyla*, respectively. (A) Flower and fruit morphology of the *Au. canescens* complex. (B) Gene density across the chromosomes. (C) Repeat composition. (D) Single nucleotide polymorphisms, multiple nucleotide polymorphisms, insertion, deletion and indel mutations across the genome.

We analysed the raw reads from three accessions of *Au. canescens* subsp. *canescens* and three accessions of *Au. macrostyla* originating from wild populations of the taxa (Fig. 1A). Each assembly was iteratively mapped and compared, and high-quality genome assembly was included in the post-assembly analysis. In total, *Au. canescens* subsp. *canescens* samples had 30 Gb (~40×), and *Au. macrostyla* samples had 29 Gb (~40×) of paired-end sequencing reads **[see****Supporting Information—Table S1****]**. All paired-end reads were used for the *de novo* assembly. Using the SPAdes software, 165 and 157 Mbp, respectively, of the draft genome of *Au. canescens* subsp. *canescens* and *Au. macrostyla* were assembled. The scaffold N50 sizes were 19.70 and 18.90 kbp and the longest contigs were 29 kbp and 28 kbp in *Au. canescens* subsp. *canescens* and *Au. Macrostyla*, respectively. BUSCO was employed to check the completeness of the draft assemblies, which detected 90 % of *Au. canescens* subsp. *canescens,* and 88 % of *Au. macrostyla* to be embryophyte genes in the two assemblies. In addition, short reads were mapped onto each corresponding assembly to evaluate the accuracy of the assemblies, and the mapping success was 98.4 % for *Au. canescens* subsp. *canescens* and 97.7 % for *Au. macrostyla* (Table 1; **see****Supporting Information—Fig. S2**).

**Table 1.** Assembly statistics of *Aubrieta canescens* subsp. *canescens* and *Au. macrostyla*.

Assembly statistics	*Au. canescens* subsp. *canescens*	*Au. macrostyla*
Assembly strategies	Iterative and *de novo*	Iterative and *de novo*
Number of scaffolds	98	105
Longest scaffold (kb)	29	28
Guanine-cytosine content (%)	34.63	34.85
Mapping accuracy (%)	98.4	97.7
Complete BUSCOs percentage (%)	90	88
N50 (kb)	19.7	18.9
N75 (kb)	17.8	16.6
Assembled genome size (Mb)	165.0	156.9
Assembled contig numbers	818.200	647.738
Number of nucleotides per 100 kb	1862.51	1821.26

### Gene model prediction and gene density

Gene model prediction was performed using the assembled scaffolds (Phred quality score > Q20 and >15× coverage). *Aubrieta canescens* subsp. *canescens* had a total of 32 425 coding DNA sequences, whereas *Au. macrostyla* had a total of 31 372 genes. A total of 417 sequences in *Au. canescens* subsp*. canescens* were found in the unplaced scaffolds of the chromosomes. Similarly, 393 sequences were found for *Au. macrostyla*. The highest gene density (20 % more than others) was observed on chromosome 8, followed by chromosome 4 in the two taxa (Fig. 1B; Table 2). Genomic profiling of the raw data was compared to evaluate heterozygosity, and the results showed that *Au. macrostyla* has a much higher genome heterozygosity (~2.5×), which is considered to involve more tandem sequences and gene models, than that of *Au. canescens* subsp. *canescens* (~0.4×, **see****Supporting Information—Fig. S1**; Tables 2 and 3) (Vurture *et al.* 2017).

**Table 2.** Chromosomal organization of gene models in *Aubrieta canescens* subsp. *canescens* and *Au. macrostyla*.

Chromosome	Gene model numbers of *Au. canescens* subsp. *canescens*	Gene model numbers of *Au. macrostyla*
Chr 1	3.989	3.875
Chr 2	2.892	2.698
Chr 3	3.960	4.033
Chr 4	4.503	4.295
Chr 5	3.441	3.306
Chr 6	3.400	3.242
Chr 7	3.971	3.686
Chr 8	5.852	5.844

**Table 3.** Repetitive sequences in *Aubrieta canescens* subsp. *canescens*, and *Au. macrostyla* genome assembly.

	Repeat class	Repeat subclass	Repeat size of *Au. canescens* subsp*. canescens* (bp)	Repeat size of *Au. macrostyla* (bp)
Retrotransposons			11 644 448	9 586 050
	SINEs		446 655	440 701
	LINEs		2 915 226	2 741 343
		L1/CIN4	2 910 180	2 735 455
	Long terminal repeat elements		8 282 567	6 404 006
		Ty1/Copia	3 096 604	2 691 881
		Gypsy/DIRS1	4 957 195	3 510 223
DNA transposons			5 110 621	4 343 654
		hobo-Activator	1 240 611	1 130 175
		Tc1-IS630-Pogo	1 229 977	964 432
		Tourist/Harbinger	526 370	469 081
Small RNA			485 668	481 167
Satellite DNA			10 493	10 493
Simple sequence repeat (microsatellite)			2 825 779	2 602 091
Low-complexity DNA			965 076	894 604
Unclassified			529 526	428 768
Interspersed repeats			17 284 595	14 358 472
Total masked TE			21 112 636 bp (12.79 %)	17 896 883 bp (11.41 %)

### Repeat composition

We analysed the repeat composition of the target *Aubrieta* taxa to infer the number of repetitive sequences in their genomes. In our whole-genome assembly, TEs comprised approximately 12.79 % and 11.41 % of the assembled parts of the genomes of *Au. canescens* subsp. *canescens* and *Au. macrostyla*, respectively. These sequences were divided into five major repeat classes: retroelements (55 % in *Au. canescens* subsp. *canescens* and 53 % in *Au. macrostyla*), DNA transposons (24 % in *Au. canescens* subsp. *canescens* and 24 % in *Au. macrostyla*), microsatellites (simple sequence repeats) (13 % in *Au. canescens* subsp. *canescens* and 15 % in *Au. macrostyla*) and low-complexity DNAs (5 % in *Au. canescens* subsp. *canescens* and 5 % in *Au. macrostyla*), and small RNAs (2 % in *Au. canescens* subsp. *canescens* and 3 % in *Au. macrostyla*) (Fig. 1C; Table 3; **see****Supporting Information—Table S3**).

Among these two taxa, the repeat content of the *Au. canescens* subsp. *canescens* genome was higher than that of *Au. macrostyla* genome (Fig. 1C). Specifically, the number of long terminal repeat elements was much higher in *Au. canescens* subsp. *canescens.* Among the classified repeat elements in the target taxa, Tourist/Harbinger made the lowest contribution to DNA transposons, followed by short interspersed nuclear elements (SINEs) in retrotransposons.

In terms of TEs, *Au. canescens* subsp. *canescens* had a higher percentage of class 1 transposon elements (34 %) than *Au. macrostyla* (32 %). Although the activity of long terminal repeat retrotransposons elements in the organism varies according to their interaction with the host genome, it can be significantly effective in the diversification of plants. The two taxa had the same percentage of class 2 transposons, *Au. canescens* subsp*. canescens* (38 %) and *Au. macrostyla* (38 %), whereas their length was 2 % and 7 % higher throughout the genome compared to *A. thaliana* (Kaul *et al.* 2000). In *Brassica oleracea*, which shares a common ancestor with *A. thaliana* that diverged around 15–20 million years ago (mya) (Yang *et al.* 1999), the proportion of mobile elements represents approximately 40 % of the entire genome (Chiu *et al.* 2010; Jiang and Ramachandran 2013). A comparison of the whole-genome sequence of the *Au. canescens* with the high-quality assembly of *A. alpina* revealed that the related species, *A. alpina*, contains a higher percentage (approximately 25 %) of TEs (Willing *et al.* 2015). Mobile elements play an important role in genomic and chromosomal evolution (Zhao *et al.* 2013; Cheng *et al.* 2016; Zhang *et al.* 2020) and more recently several attempts have also been made to construct phylogenies using TE abundance as an informative character (Dodsworth *et al.* 2015; Vitales *et al.* 2020; Beric *et al.* 2021). In addition, the repeat content is known to interfere with gene function and may result in the formation of variants that are responsible for phenotypic changes (Liu *et al.* 2020). A comprehensive analysis of repetitive elements in our novel genome would provide valuable knowledge on morphological variations and evolution of the *Au. canescens* complex and its phylogenetic relationships in *Aubrieta* and Arabideae.

### Variant identification

In the genome-wide variant exploration of the species, the minimum quality score of reads was 30.0 **[see****Supporting Information—Figs S3****and****S4****]**. A total of 1 108 387 single nucleotide polymorphisms (SNPs) were found in *Au. canescens* subsp*. canescens*, representing 72 % of the variants found in the entire genome, and mostly being found on chromosomes 1 and 7. A total of 434 714 SNPs were identified in *Au. macrostyla* corresponding to 76 % of the total variants. In chromosome 2, rare but long base-pair variants were observed, whereas in chromosome 4 and chromosome 8, variants with dense but longer base pairs than the average distribution were detected **[see****Supporting Information—Figs S5****and****S6****]**. In *Au. canescens* subsp*. canescens* the insertion/deletion ratio was 1.10, and the SNP transitions/transversions ratio was 1.39. In contrast, *Au. macrostyla* had an insertion/deletion ratio of 0.93 and a SNP transitions/transversions ratio of 1.42. In addition, the highest ratio of SNPs, MNPs, insertion, deletion and indel mutation content (14.2 % of all variants) was determined in *Au. canescens* subsp*. canescens*, whereas *Au. macrostyla* genome had the lowest variant content (Fig. 1D).

### Evolutionary aspects

**Figure 2.**
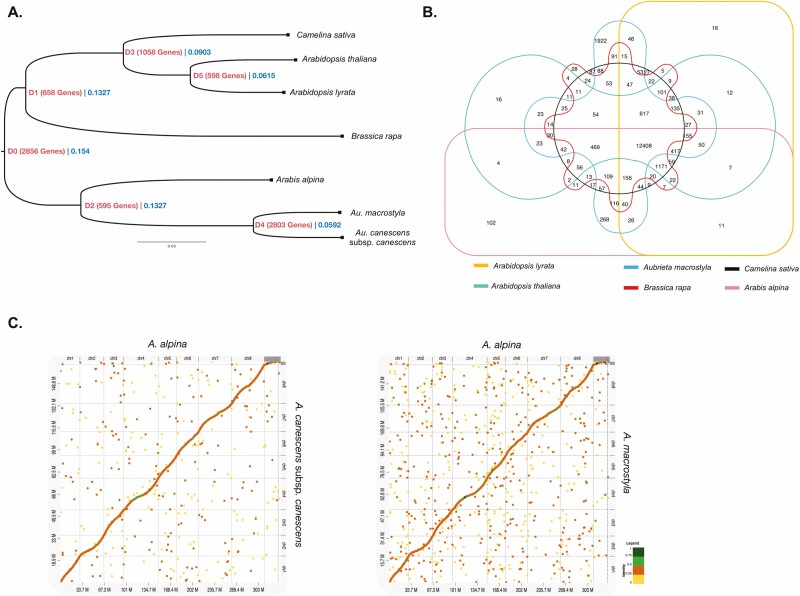
Genome evolution and comparative genomic analyses. (A) Homology-based phylogenetic tree of the *Au. canescens* complex and other Brassicaceae taxa. Gene numbers in nodes represent gene duplication events, and decimals in blue indicate node age. (B) Venn diagram showing the number of orthologous genes in *Au. canescens* subsp. *canescens* along with *Arabis alpina*, *Brassica rapa*, *Camelina sativa*, *Arabidopsis thaliana*, *Arabidopsis lyrata* and *Au. macrostyla*. (C) Relationships of syntenic genes between *A. alpina* and *Au. canescens* subsp*. canescens*, and *Au. macrostyla.* According to the identity scale, highly matched DNA sequences are indicated by dark green to light green dots (50–100 %), moderately matched sequences by orange dots (25–50 %) and poorly matched sequences by yellow dots (0–25 %). Sequences that did not match are shown in white.

We compared gene data originating from the *Aubrieta* taxa with the related genome data of core Brassicaceae species (Fig. 2). Based on the phylogenomic tree, two representatives of Clade A, *Camelina sativa* and *Arabidopsis lyrata*, and members of Clade B, *Brassica rapa* species differentiated from *A. thaliana*, are well supported (Fig. 2A). Notably, the ratio of syntenic gene pairs (77–80 %) of *B. rapa* to that of *A. thaliana* and *A. lyrata* support this topology in previous studies (Cheng *et al.* 2012). Moreover, the *Au. canescens* complex showed close affinity with *A. alpina*, a genus in Clade B. These results provide evidence that the *Aubrieta* genus must be classified as a member of Clade B.

Previously, Karl *et al.* (2013) reported that 15 species of the genus *Aubrieta* diverged from the Arabideae tribes approximately 2.7–5.2 mya in the Eastern Mediterranean region. Likewise, orthologous genes indicate that *Aubrieta* species differentiated from *A. alpina* later, at 1.3–2.0 mya. Based on the homologous gene clusters obtained from OrthoFinder, we asserted that 2803 paralog genes distinguish *A. alpina* from the *Aubrieta* species. We consider that paralogs with a similar number of genes distinguish *Au. macrostyla* well from *Au. canescens*.

The percentage of assigned gene clusters in *Au. canescens* subsp. *canescens* and *Au. macrostyla* was 96.5 % and 95.4 %, respectively, within the 365 251 genes obtained from OrthoFinder. The number of gene clusters common to all species was 7078, whereas the number of single-copy gene clusters was 295. We consider that *Au. macrostyla* evolved before *Au. canescens* subsp. *canescens* and belongs to a different branch in terms of orthologs. The highest number of genes in a species-specific gene cluster was detected for *Au. canescens* subsp. *canescens* (244), followed by *Au. macrostyla* (232). Two taxa shared the most orthologous genes with perennial *A. alpina* (14 356) and *A. lyrata* (14 336) species (Fig. 2B).

Syntenic gene identification was performed between *A. alpina* (39 815 annotated proteins) and *Au. canescens* subsp*. canescens* (32 425) and *Au. macrostyla* (31 372). Prior to syntenic gene determination, duplicated tandem genes were removed from the genome*. Arabis alpina* returned 28 400 genes that showed synteny with 32 209 *Au. canescens* subsp*. canescens* and 28 982 *Au. macrostyla* genes. After eliminating redundant sequences, we identified syntenic gene pairs for each taxon. Although there were no remarkable differences between the species, the synteny ratio of *A. alpina* to *Au. canescens* subsp*. canescens* and *Au. macrostyla* was 46 % and 45 %, respectively. Most of the tandem arrays on the chromosomes 1, 3 and 8 of *A. alpina* showed strict synteny with the *Au. canescens* complex (Fig. 2C; Tables 4 and 5). These results show that chromosomes 4 and 7 are less informative in the syntenic gene arrangement, regardless of assembly size.

**Table 4.** Syntenic orthologous genes between *Arabis alpina* to *Aubrieta canescens* and *Au. macrostyla*. *Number of overlapped sequences.

Syntenic orthologs to *A. alpina*	*A. alpina* vs. *Au. canescens* subsp. *canescens*	*A. alpina* vs*. Au. macrostyla*
Chr1 (arrays* | bp | genes)	377.339 | 7.194.247 | 1.926	424.768 | 7.540.313 | 1.788
Chr2 (arrays* | bp | genes)	485.449 | 5.466.368 | 1.414	423.693 | 4.799.368 | 1.230
Chr3 (arrays* | bp | genes)	296.393 | 7.323.310 | 1.853	274.384 | 6.922.344 | 1.797
Chr4 (arrays* | bp | genes)	628.093 | 7.719.562 | 1.544	610.820 | 7.587.290 | 1.563
Chr5 (arrays* | bp | genes)	416.086 | 5.828.293 | 1.470	390.924 | 5.661.399 | 1.599
Chr6 (arrays* | bp | genes)	303.688 | 5.863.068 | 1.707	275.829 | 5.454.359 | 1.597
Chr7 (arrays* | bp | genes)	459.382 | 6.696.666 | 1.524	443.276 | 6.433.374 | 1.436
Chr8 (arrays* | bp | genes)	676.307 | 10.542.223 | 1.717	647.019 | 10.509.024 | 1.815

**Table 5.** Comparative homology of *Arabis alpina* and studied taxa *Aubrieta canescens* subsp*. canescens*, and *Au. macrostyla.*

Taxa	Number of genes	Number of removed tandem genes	Orthologous genes to *A. alpina*	Non-orthologs to *A. alpina*	Orthologous-based syntenic genes to *A. alpina* (identity >70 %)
*Au. canescens* subsp*. canescens*	32.425	216	19.373	13.052	13.155
*Au. macrostyla*	31.372	2.390	20.177	11.195	12.825

The ratio of orthologous-based syntenic genes of *A. alpina* to *Au. canescens* subsp. *canescens* (13 155/19 373) was 68 %, whereas that of *A. alpina* to *Au. macrostyla* (12 825/20 177) was 63.5 % (Table 5). We can consider the existence of these genes in additional arrays on chromosomes 1, 3, 6 and 8 in *Au. canescens*, whereas these genes were mostly ordered on chromosomes 1, 3 and 8 in *Au. macrostyla* (Table 4). Therefore, we can assume that *Au. macrostyla* has a clear genome-wide distinction, with orthologous domains that differ from those of *A. alpina* and *Au. canescens*.

The availability of genome data for *Aubrieta* taxa will enable us to analyse the population genomics within the genus *Aubrieta* in further studies. Additionally, this study will facilitate the elucidation of the *Au. canescens* complex, including *Au. canescens* subsp*. cilicica*. As a result, our data provide a significant contribution to the *Aubrieta* genome resource and novel insights into the evolution of Arabideae plants. The genome sequence produced here may help improve the potential horticultural value of *Aubrieta* taxa; however, a high-quality genome is needed for use in plant breeding programs and horticulture.

## Supporting Information

The following additional information is available in the online version of this article—

**Table S1.** Sequencing reports of total six populations of *Aubrieta canescens* complex.

**Table S2.** Genomes used for comparative analysis in Brassicaceae family.

**Table S3.** Summary of repetitive elements in *Aubrieta canescens* subsp. *canescens* and *Au. macrostyla* genome.

**Figure S1.** BUSCO assessment for the taxa *Aubrieta canescens* subsp. *canescens* and *Au. macrostyla*.

**Figure S2.** Genomic profile plots of (A) *Aubrieta canescens* complex raw reads and (B) *Au. macrostyla* raw reads.

**Figure S3.** Quality score of bases obtained in variant identification. Selected bases are greater than 30 Phred score on all accessions.

**Figure S4.** Estimated coverage of bases used in variant identification. Minimum 15× base coverage was selected for determining polymorphisms on all accessions.

**Figure S5.** SNP density of *Aubrieta canescens* subsp. *canescens* across the chromosomes. The contig size of variants is kilobase pairs. Although the longest contigs were observed on chromosome 7, SNPs were observed in long contigs in chromosomes 3, 4 and 8 as well.

**Figure S6.** SNP density of *Aubrieta macrostyla* across the chromosomes. The contig size of variants is kilobase pairs. The longest contigs (30 kb) containing SNPs were observed on chromosome 2. Also, longer-than-average contigs (18 kb) were detected on chromosomes 4 and 7.

plac035_suppl_Supplementary_Material

## Data Availability

The raw data of *Aubrieta canescens* subsp. *canescens* and *Au. macrostyla* have been submitted to the NCBI SRA database under the Bioproject Numbers PRJNA789858 and PRJNA790745.

## Notes

**Evolution & Diversity. Chief Editor**: Jeremy Beaulieu
