# Supplementary material for: Genome-wide characterization of two Aubrieta taxa: Aubrieta canescens subsp. canescens and Au. macrostyla (Brassicaceae)
Source: AoB Plants. 2022 Sep 10;14(5):plac035. doi: 10.1093/aobpla/plac035 (PMC9521481; doi:10.1093/aobpla/plac035)
Supplement: plac035_suppl_Supplementary_Material [file plac035_suppl_supplementary_material.pdf]

**Tables**

Table S1. Sequencing reports of total 6 populations of *Aubrieta canescens* complex.

| **Samples** | **Sequencing Platform** | **Insert Size (bp)** | **Total Reads (bp)** | **Sequence Coverage * (X)** |
|---|---|---|---|---|
| *A. canescens* subsp. *canescens* - AAD20168 | BGI DNB SEQ - 500WGS | 150 × 2 | 121382020 | 50.0 |
| *A. canescens* subsp. *canescens* - AAD20345 | BGI DNB SEQ - 500WGS | 150 × 2 | 311056036 | 30.1 |
| *A. canescens* subsp. *canescens* - AAD19792 | Illumina NovaSeq 6000 | 150 × 2 | 42108206 | 10.0 |
| *A. macrostyla* - AAD20146-1 | BGI DNB SEQ - 500WGS | 150 × 2 | 107455344 | 33.9 |
| *A. macrostyla*- AAD20146-2 | BGI DNB SEQ - 500WGS | 150 × 2 | 314768940 | 46.3 |
| *A. macrostyla* - AAD20051 | Illumina NovaSeq 6000 | 150 × 2 | 42381390 | 15.0 |

Table S2. Genomes used for comparative analysis in Brassicaceae family.

| **Species** | **Version** | **Data source/ GenBank accession number** |
|---|---|---|
| *Arabidopsis lyrata* | 1.0 | https://phytozome.jgi.doe.gov |
| *Arabidopsis thaliana* | Tair10 | https://www.arabidopsis.org |
| *Brassica rapa* | 3.0 | http://brassicadb.cn/#/ |
| *Camelina sativa* | release-52 | https://plants.ensembl.org/Camelina_sativa/Info/Index |
| *Arabis alpina* | 5.1 | http://www.arabis-alpina.org |

Table S3. Summary of repetitive elements in *A. canescens* subsp. *canescens* and *A. macrostyla* genome.

| | Repeat class | Repeat subclass | *A. canescens* subsp. *canescens* (Number of elements) | *A. macrostyla* (Number of elements) |
|---|---|---|---|---|
| Retrotransposons | | | 24061 | 20273 |
| | SINEs | | 3143 | 3110 |
| | LINEs | | 4464 | 4167 |

| | | | | |
|---|---|---|---|---|
| | | L1/CIN4 | 4426 | 4124 |
| | LTR elements | | 16454 | 12996 |
| | | Ty1/Copia | 4980 | 4214 |
| | | Gypsy/DIRS1 | 10860 | 8171 |
| DNA transposons | | | 23172 | 20326 |
| | | hobo-Activator | 5987 | 5384 |
| | | Tc1-IS630-Pogo | 6371 | 5359 |
| | | Tourist/Harbinger | 1713 | 1657 |
| Small RNA | | | 3615 | 3615 |
| Satellite DNA | | | 71 | 44 |
| SSR (Microsatellite) | | | 70136 | 65309 |
| Low Complexity DNA | | | 18453 | 17233 |
| Total masked TE | | | 21112636 bp (12.79 %) | 17896883 bp (11.41 %) |

**Data analysis**

Raw reads are filtered of adapters and low-read sequences by SOAPnuke  v2.1.6 algorithm (Chen et al., 2018). The parameters used are as follows: -n 0.01 -l 20 -q 0.3 -A 0.25 --cutAdaptor -Q 2 -G --polyX 50 --minLen 150.

Raw data (in fastq format) were analyzed with the FastQC (Andrew, 2010) software to perform quality control procedures before bioinformatics analysis.

Missmatch base pairings that may be caused by the sequencing machine of the genomic data were removed from the genome with the Karect (Allam et al., 2015) software before assembly. Options for Karect software was: -matchtype=hamming and -celltype=haploid.

Kmerginie (Chikhi & Medvedev, 2014) software was used to determine the optimum number of k-mers to screen the entire genome for genome assembly. A low number of K-mer lengths may increase the likelihood of base mismatches. Therefore, when choosing the number of k-mers, it should be considered that it is a value that best covers the genome and is of sufficient length. After the optimum number of k-mers was determined, the filtered genome data was de novo assembly with the Python-based SPAdes v3.15(Bankevich et al., 2012) program. Since error readings are minimized before assembly, only the assembly feature of the program is preferred. The parameters used are as follows:

spades.py -1 file_name_1.fastq.gz -2 file_name_2.fastq.gz -t 64 -k 85 --careful --only-assembler

**Figures**

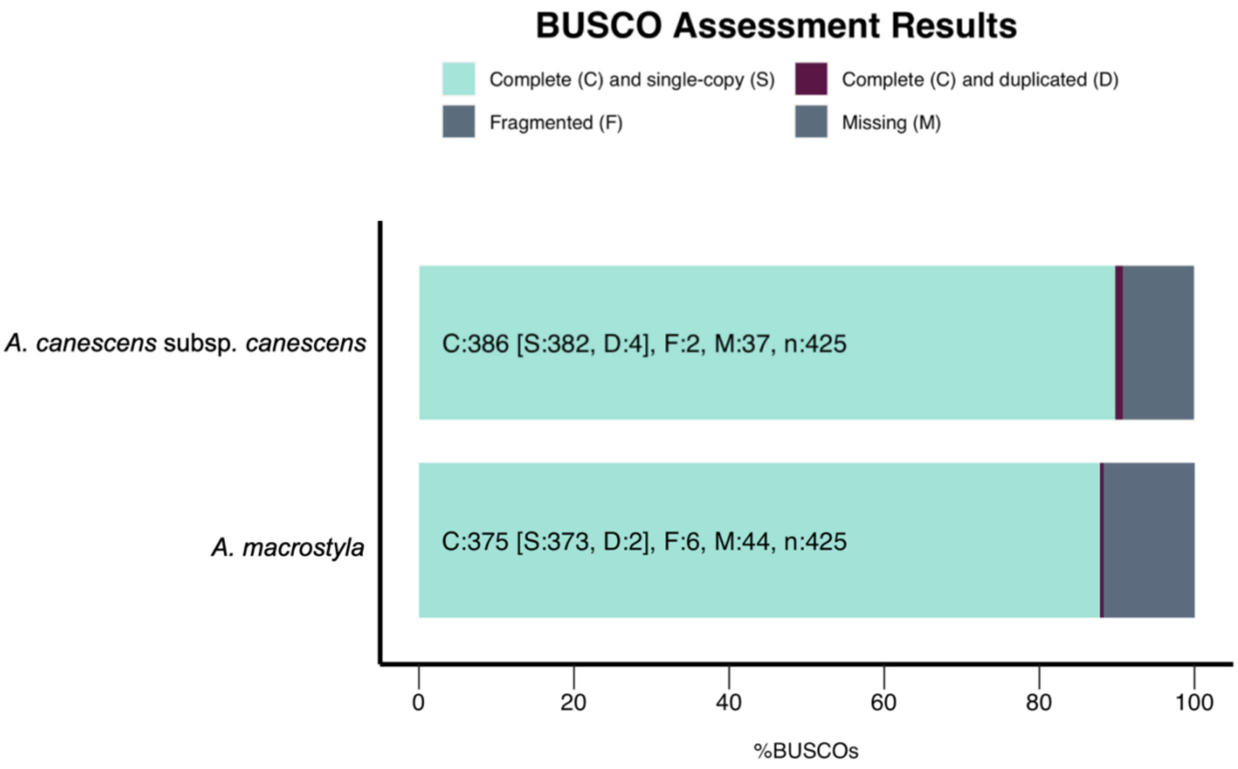


Figure S1. BUSCO assessment for the taxa *Aubrieta canescens* subsp. *canescens* and *A. macrostyla*.

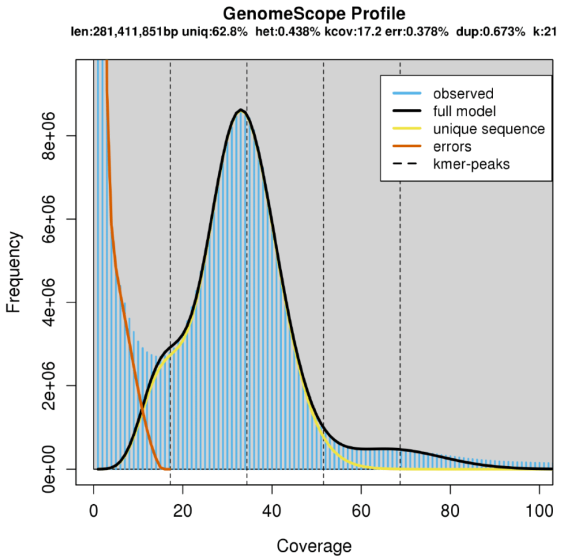


*A. canescens* subsp. *canescens*

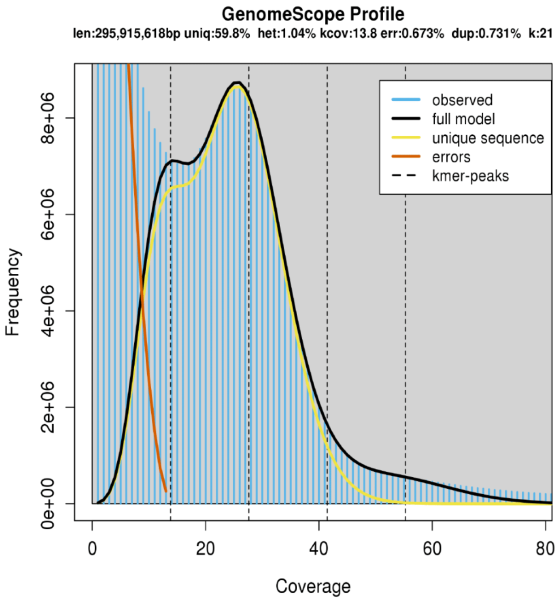


*A. macrostyla*

Figure S2. A) Genomic profile plots of *A. canescens* complex raw reads.

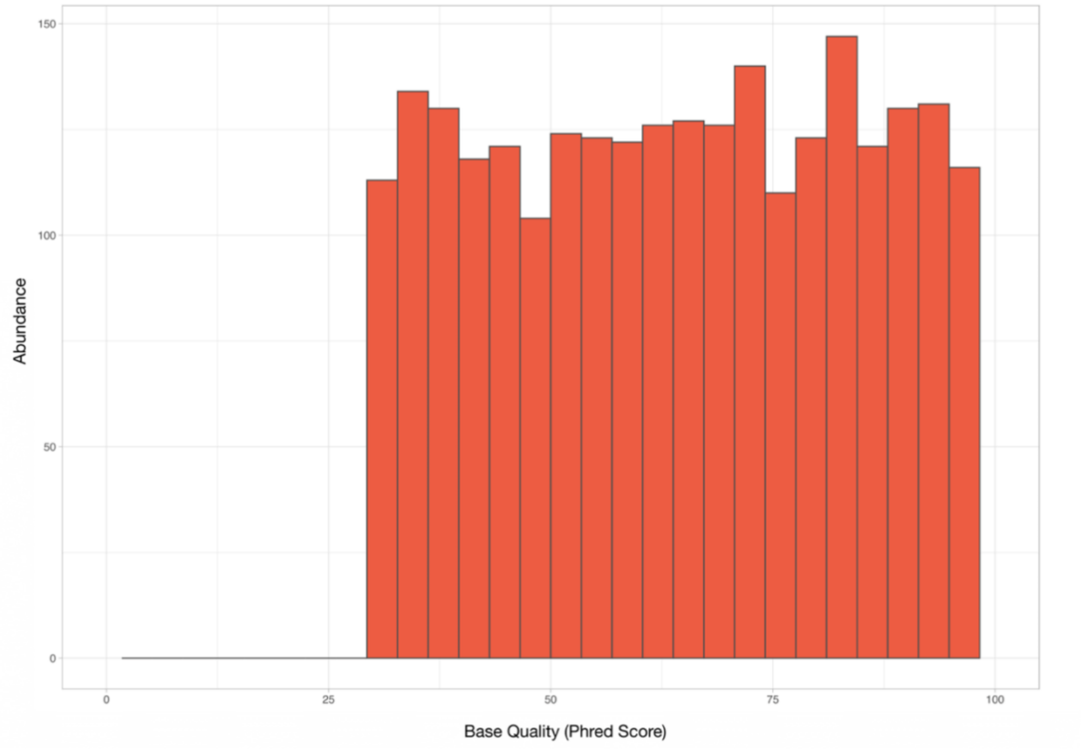


Figure S3. Quality score of bases obtained in variant identification. Selected bases are greater than 30 phred score on all accessions.

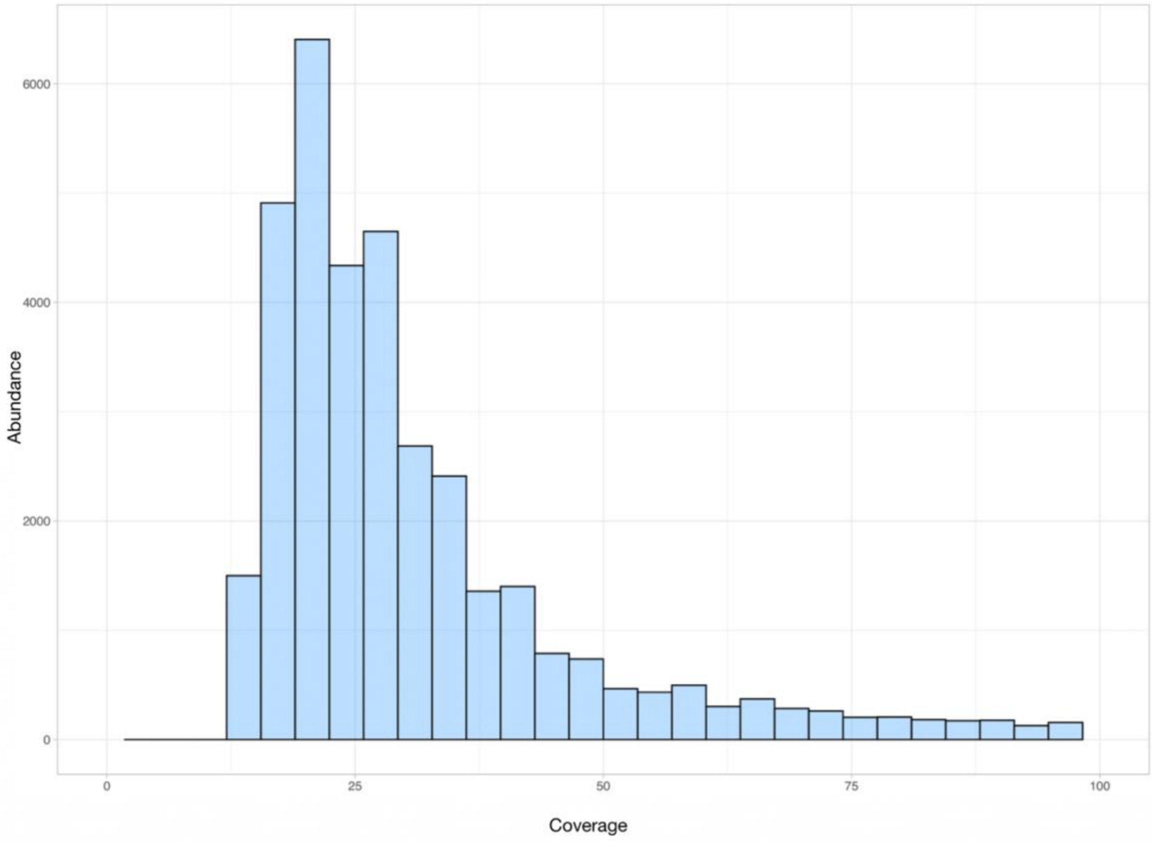


Figure S4. Estimated coverage of bases used in variant identification. Minimum 15X base coverage was selected for determining polymorphisms on all accessions.

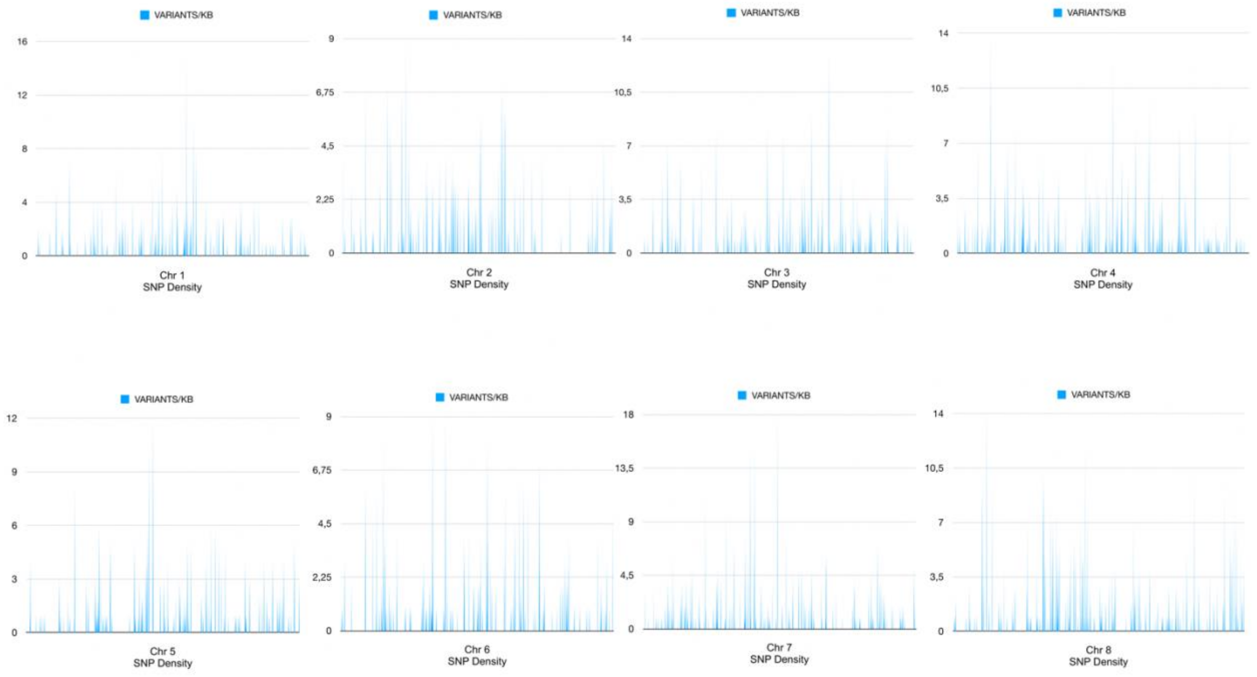


Figure S5. SNP density of *Aubrieta canescens* subsp. *canescens* across the chromosomes. The contig size of variants is kilobase pairs. Although the longest contigs were observed on chromosome 7, SNPs were observed in long contigs in chromosomes 3, 4, and 8 as well.

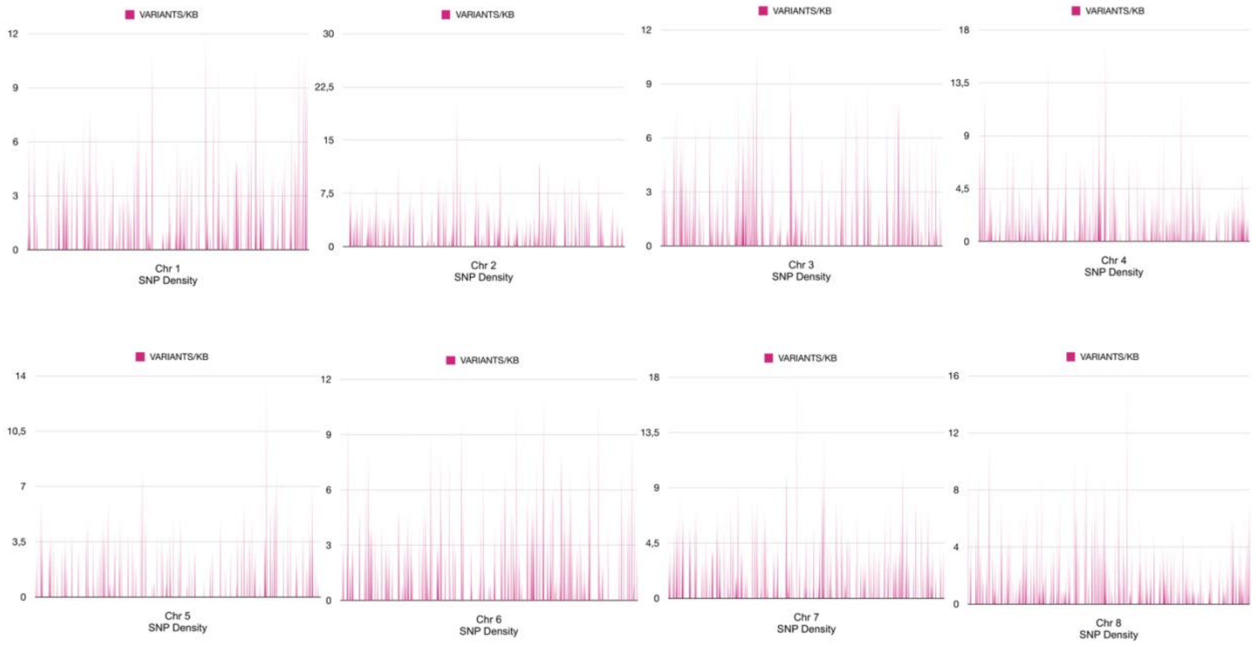

Figure S6. SNP density of *Aubrieta macrostyla* across the chromosomes. The contig size of variants is kilobase pairs. The longest contigs (30 kb) containing SNPs were observed on chromosome 2. Also, longer-than-average contigs (18 kb) were detected on chromosomes 4 and 7.

## References


Allam, A., Kalnis, P., & Solovyev, V. (2015). Karect: accurate correction of substitution, insertion and deletion errors for next-generation sequencing data. *Bioinformatics*, *31*(21), 3421–3428. https://doi.org/10.1093/bioinformatics/btv415

Andrew, S. (2010). *FastQC: A quality control analysis tool for high throughput sequencing data*. https://github.com/s-andrews/FastQC

Bankevich, A., Nurk, S., Antipov, D., Gurevich, A. A., Dvorkin, M., Kulikov, A. S., Lesin, V. M., Nikolenko, S. I., Pham, S., Prjibelski, A. D., Pyshkin, A. v., Sirotkin, A. v., Vyahhi, N., Tesler, G., Alekseyev, M. A., & Pevzner, P. A. (2012). SPAdes: A new genome assembly algorithm and its applications to single-cell sequencing. *Journal of Computational Biology*, *19*(5), 455–477. https://doi.org/10.1089/cmb.2012.0021

Chen, Y., Chen, Y., Shi, C., Huang, Z., Zhang, Y., Li, S., Li, Y., Ye, J., Yu, C., Li, Z., Zhang, X., Wang, J., Yang, H., Fang, L., & Chen, Q. (2018). SOAPnuke: A MapReduce acceleration-supported software for integrated quality control and preprocessing of high-throughput sequencing data. *GigaScience*, *7*(1), 1–6. https://doi.org/10.1093/gigascience/gix120

Chikhi, R., & Medvedev, P. (2014). Informed and automated k-mer size selection for genome assembly. *Bioinformatics*, *30*(1), 31–37. https://doi.org/10.1093/bioinformatics/btt310
